# Immunogenic profile of a plant-produced nonavalent African horse sickness viral protein 2 (VP2) vaccine in IFNAR^-/^^- ^mice

**DOI:** 10.1371/journal.pone.0301340

**Published:** 2024-04-16

**Authors:** Martha M. O’Kennedy, Robyn Roth, Karen Ebersohn, Lissinda H. du Plessis, Sipho Mamputha, Daria A. Rutkowska, Ilse du Preez, Jan A. Verschoor, Yolandy Lemmer

**Affiliations:** 1 Council for Scientific and Industrial Research (CSIR), Chemical Cluster, Pretoria, South Africa; 2 Department of Veterinary Tropical Diseases, University of Pretoria, Pretoria, South Africa; 3 Centre of Excellence for Pharmaceutical Sciences (PharmacenTM), North-West University, Potchefstroom, South Africa; 4 Department of Biochemistry, University of Pretoria, Pretoria, South Africa; Universidad Nacional de la Plata, ARGENTINA

## Abstract

A safe, highly immunogenic multivalent vaccine to protect against all nine serotypes of African horse sickness virus (AHSV), will revolutionise the AHS vaccine industry in endemic countries and beyond. Plant-produced AHS virus-like particles (VLPs) and soluble viral protein 2 (VP2) vaccine candidates were developed that have the potential to protect against all nine serotypes but can equally well be formulated as mono- and bi-valent formulations for localised outbreaks of specific serotypes. In the first interferon α/β receptor knock-out (IFNAR^-/-^) mice trial conducted, a nine-serotype (nonavalent) vaccine administered as two pentavalent (5 μg per serotype) vaccines (VLP/VP2 combination or exclusively VP2), were directly compared to the commercially available AHS live attenuated vaccine. In a follow up trial, mice were vaccinated with an adjuvanted nine-serotype multivalent VP2 vaccine in a prime boost strategy and resulted in the desired neutralising antibody titres of 1:320, previously demonstrated to confer protective immunity in IFNAR^-/-^ mice. In addition, the plant-produced VP2 vaccine performed favourably when compared to the commercial vaccine. Here we provide compelling data for a nonavalent VP2-based vaccine candidate, with the VP2 from each serotype being antigenically distinguishable based on LC-MS/MS and ELISA data. This is the first preclinical trial demonstrating the ability of an adjuvanted nonavalent cocktail of soluble, plant-expressed AHS VP2 proteins administered in a prime-boost strategy eliciting high antibody titres against all 9 AHSV serotypes. Furthermore, elevated T helper cells 2 (T_h_2) and T_h_1, indicative of humoral and cell-mediated memory T cell immune responses, respectively, were detected in mouse serum collected 14 days after the multivalent prime-boost vaccination. Both T_h_2 and T_h_1 may play a role to confer protective immunity. These preclinical immunogenicity studies paved the way to test the safety and protective efficacy of the plant-produced nonavalent VP2 vaccine candidate in the target animals, horses.

## Introduction

African horse sickness (AHS) is an acute non-contagious infectious viral disease of equids transmitted by *Culicoides spp*. biting midges and lethal to susceptible equids. AHS is a World Organisation for Animal Health (WOAH), formerly the Office International des Epizooties (OIE) notifiable disease. The causal agent is the African horse sickness virus (AHSV), within the genus *Orbivirus* of the *Reoviridae* family [1]. Nine different serotypes of AHSV have been identified [2]. Though protective immunity is considered long-lived it is serotype-specific [3] with limited cross neutralisation [4]. AHS is endemic to Sub-Saharan Africa but outbreaks in America or Europe have been feared by the horse industry for decades due to the competency of the native *Culicoides* vectors in both continents [5]. Vaccination remains the most important method to curtail AHS spread [4] but also requires control of animal movements as well as prevention of *Culicoides* bites [3, 6]. Effective treatment of the disease is still evasive. Since 1974, the commercially available live-attenuated vaccine (LAV), lacking serotypes 5 and 9, has been extensively used in South Africa and other African countries but is not licenced in non-endemic countries. Major concerns include reversion to virulence, and reassortment between LAVs and field viruses leading to more pathogenic virus variants. Although inactivated vaccines are generally considered safe, incomplete inactivation of the virus is a threat to animal health in a similar way to insufficient attenuation [7].

Numerous alternative vaccine initiatives to combat AHS in endemic countries and beyond the borders of Africa have previously been described [8] but none are commercially available and the initiative to develop modern safe and efficacious subunit vaccines remain elusive. One of the more promising subunit vaccines being developed are plant-produced virus-like particles (VLPs) as these are robust protein scaffolds exhibiting well-defined geometry and uniformity that mimic the overall structure of the native virions. VLPs lack the viral genome and are thus considered safe while at the same time being antigenically indistinguishable from the virus from which they were derived [9]. The AHSV virion is a triple layered particle formed by the outer capsids (VP2 and VP5), the middle layer (VP7), and the inner shell (VP3, subcore) [10]. Plant-produced, homogenous AHSV-5 VLP-based vaccines, administered in a prime-boost regime, resulted in high antibody titres being elicited in guinea pigs and horses [11, 12], but relatively low titres in horses when they were vaccinated with the chimaeric AHSV-6 VLPs [13] most likely due to the poor assembly of VLPs of serotype 6 at the time. Subsequently, protective immunity was demonstrated with fully assembled chimaeric AHSV-5 VLPs in IFNAR^-/-^ mice [8]. Due to the complexity of successfully assembling four capsid proteins into a triple layer, to form AHS VLPs of each serotype, assembling all nine AHS serotypes individually remained challenging. Thus, complementary to the VLPs of selected serotypes which assembled with ease, VP2 proteins were harnessed to compile the nine serotypes prime-boost formulation. The ease of plant production, partial purification, and concentration of the soluble VP2 proteins, also facilitated an efficient and cost-effective nonavalent vaccine formulation.

VP2 is the major determinant inducing serotype specific neutralising antibodies (nAbs) [7, 14] and correct display of essential epitopes in their native conformation is imperative. It is well established that full length soluble VP2 is sufficient to induce protective immunity in mice [8, 15] and horses [5] whilst insoluble VP2 results in poor immunogenicity [4]. Humoral immunity plays a pivotal role in protection against AHSV [3, 16, 17] but other mechanisms such as antibody-dependent cellular cytotoxicity (ADCC) or other cell-mediated responses cannot be ignored as contributing factors [5, 18]. Indeed, de la Poza [19] suggested that conserved T-cell epitopes that display cytotoxic T cell (CTL) activity can contribute to the generation of protective multivalent vaccines against AHSV.

A new generation, safe and efficacious vaccine to protect against African horse sickness has been elusive for more than twenty years. To this end, CSIR sought to develop vaccine candidates against African horse sickness to penetrate the global market but also cost effective to serve South African local communities and other African low- and middle-income countries (LMICs). Once again Interferon α/β receptor knock-out (IFNAR^-/-^) mice served as a cost-effective model to precede horse trials in vaccine candidate testing. It was previously demonstrated that a plant-produced monovalent AHSV-5 VP2 vaccine eliciting a titre of 1:320, conferred protective immunity in IFNAR^-/-^ mice [8]. Here, two consecutive immunogenicity studies were conducted in IFNAR^-/-^ mice, to validate the plant-produced multivalent immunogens directly with the commercially available LAV vaccine. First, either a combination of plant-produced VLPs and soluble VP2 antigens; or exclusively VP2 antigens were formulated for pentavalent vaccinations. It was evident from the first mice trial, that each serotype required a booster vaccination and subsequently mice were vaccinated with the VP2 antigens of all nine serotypes (nonavalent) in a prime boost regime which then elicited high neutralising antibody titres ranging from 1:112 to 1:320. In addition, to avoid licence agreements for the use of glyco-engineered *Nicotiana benthamiana* [20] used in the previous study [8] and thereby simplifying commercialisation, here the vaccine products were produced in wild type *N*. *benthamiana*.

## Materials and methods

### Cloning of constructs

Cloning was described in detail previously [8]. Briefly, the plant expression vector, pEAQ-HT, harbours the individual genes encoding AHS VP2^His^ fusion proteins (serotypes 1–9) as per the patent application entitled: “African horse sickness viral protein 2 (VP2) fusion proteins” (PCT/IB2023/058808).

### Production and partial purification of plant-produced VLPs

*Agrobacterium* strain AGL-1 (ATCC^®^ BAA-101^TM^) harbouring each of the constructs encoding AHSV-1 VP3/VP7, AHSV-5 VP2 and AHSV-5 VP5 proteins, adjusted to OD_600_ = 1.6 were mixed in a ratio of 2:1:1, respectively, for hand infiltration of wild type *N*. *benthamiana* plants. Double chimaeric AHSV-6 VLPs were assembled similarly as double chimaeric AHSV-5 described above but the outer shell was comprised of AHSV-6 VP2 and AHSV-6 VP5 proteins. A lysate was prepared by juicing the leaves (∼100 grams) harvested 7–8 days post infiltration in a Bicine buffer (50 mM Bicine, 20 mM NaCl pH 8.4) supplemented with protease inhibitor cocktail (Sigma P2714). Plant debris was then removed by depth filtration followed by concentration using tangential flow filtration (TFF) 100K Minimate^TM^ TFF Capsule (Pall Life Sciences). The concentration of the VLPs per ml of TFF purified lysate was determined using Optiprep (Sigma) density gradient ultracentrifugation isolation of the VLPs as described before [8].

### Production and purification of AHSV VP2^His^ proteins

Leaf material of *N*. *benthamiana* wild type plants infiltrated with AGL-1 harbouring the individual pEAQ-HT-AHSV-n-VP2^His^ (n = serotypes 1–9) constructs (OD_600_ = 1.4) were harvested (∼20–30 grams) five days post infiltration, juiced (leaf tissue:buffer ratio of 1:2) and briefly homogenise in a Bicine buffer supplemented with 0.1% (w/v) N-Lauroylsarcosine sodium salt and protease inhibitor cocktail (Sigma P2714). Centrifuge clarified supernatant was subjected to pre-packed immobilized metal ion affinity chromatography (IMAC), with nickel as the metal ion (Macherey Nagel Protino® Ni-TED 2000, Catalogue number 745120.25). Bound protein was eluted isocratically with 5 bed volumes of imidazole-containing buffer, concentrated using Vivaspin 6 columns (Sartorius, VS0601) in a swing bucket rotor (4 000 g x 15–20 minutes) and dialysed twice within 16 hours against 2 L PBS buffer (140 mM NaCl, 1.5 mM KH_2_PO_4_, 10 mM Na_2_HPO_4_, 2.7 mM KCl, pH 7.4) at 4°C prior to vaccine formulation. To coat the enzyme-linked immunosorbent assay (ELISA) plates, VP2 was further purified using Dynabeads^TM^ (Invitrogen, Catalogue number 10104D) as described before [8]. For the follow up mice trial, plant-produced VP2 of all nine serotypes were individually produced as described above however a bicine buffer was used throughout from extraction until filter sterilisation, and Zeba^TM^ Spin Desalting Columns (7K MWCO, 989892) were used instead of the dialysis procedure.

### Protein stabilising and characterisation of immunogens

D-(+)-Trehalose dihydrate (Sigma-Aldrich) (15% m/v) was added to the purified immunogens (VLPs and VP2) before filter sterilisation (0.45 μM+0.2μM Sartopore 2, Sartorius, 5441307H4). The immunogens were then characterised using sodium dodecyl sulphate polyacrylamide gel electrophoresis (SDS-PAGE), transmission electron Microscopy (TEM), LC-MS/MS based peptide sequencing and Pierce^TM^ Micro BCA protein quantification (Catalogue # 23235, Thermo Scientific) prior to vaccine formulation.

### IFNAR^-/-^ mice trial design

IFNα/βR^-/-^ mice (A129, IFNAR type 1) were bred at the Preclinical Drug Development Platform (PCDDP) at North-West University, Potchefstroom, South Africa with the necessary licence in place from B&K Universal, Marshall BioResources. Female mice (7-week old) were acclimatised under pathogen-free conditions in the biosafety level 3 (BSL-3) facility for 7 days prior to vaccination. In each trial, four groups of IFNAR^-/-^ mice (n = 6) were vaccinated intraperitoneally with 5 μg of each plant-produced VLPs/VP2 or VP2 (200 μl formulated vaccine per mice) using a fixed needle syringe (25G diameter, 16 mm length). Mice (trial 1) were prime boost vaccinated (days 0 and 14) with PBS buffer as negative control (group 1), the commercial live attenuated vaccine (LAV) of Onderstepoort Biological Products (OBP) as positive control (group 2), pentavalent vaccines consisting of VLPs/VP2 (group 3) or exclusively pentavalent VP2 vaccine (group 4). The vaccines of groups 1, 3–4 were adjuvanted with 10% Montanide GEL 01 whereas the commercial vaccine contains its own proprietary adjuvant. Mice were vaccinated with 1 μl (trial 1) or 0.2 μl (trial 2) of the LAV vaccine. Sera were collected from the mice on days 0 and 28. All sera collected were heat inactivated at 56°C for 30 minutes before transport to the designated analysis facilities. Mice (trial 2) were vaccinated as described above, however the animals in groups 3 and 4 were inoculated with 5 ug VP2 of each of the nine serotypes (nonavalent) in the primary and booster vaccine formulated in Bicine buffer. The negative control group was also inoculated with Bicine buffer (group 1) to accommodate the non-compatibility of the Nanoalum to a phosphate buffer. Groups 3 and 4 were, were adjuvanted with either Montanide Gel 01 or Nanoalum, respectively. Serum samples were collected at day 0 and 28 when the trials were terminated and analysed for serotype specific neutralising antibodies by serum neutralising tests (SNTs) and VP2-specific ELISA. All mice were euthanised by cervical dislocation on day 28, the experimental endpoint. Animals that lost more than 15% of body weight during the study, was euthanised prior to the experimental endpoint (day 28).

### Detection of antibodies against purified plant-produced VP2 by ELISA

Mice sera of all six mice were pooled in each treatment group at the trial end point (day 28) and prior to ELISA and SNT tests. MaxiSorp plates (Thermo Scientific) were coated with 1 μg VP2 per well (IMAC and Dynabeads purified) and incubated for two days at 4°C. Plates were saturated with 200 μl blocking buffer (PBS-0.05% Tween 20 and 4% casein hydrolysate) and incubated for 90 minutes. The wells were washed three times with PBS-0.05% Tween 20. Thereafter, mice sera were diluted in blocking buffer (1:200) were added and incubated for 1 h at room temperature. After three washes in PBS-0.05% Tween 20, plates were incubated for 1 h at room temperature with isotype specific reagents at 1:1000 (IgG1 or IgG2a; mouse monoclonal antibody Isotyping reagents, Sigma ISO2-1KT) for 30 minutes. After three washes, a donkey anti-goat-HRP secondary antibody (Donkey anti-goat IgG H&L (HRP) preabsorbed, ab97120, abcam) at a 1:5000 dilution was added and incubated for 1 hour. Finally, after three washes in PBS-0.05% Tween 20, the reaction was developed with 50 μl substrate 3,3′,5,5′-Tetramethylbenzidine (TMB from Sigma, T0440-100ml) per well and stopped by adding 50 μl of 3 N H_2_SO_4_. Results were expressed as optical densities (ODs) measured at 450 nm.

### Serum neutralising tests (SNTs)

A 1:5 start dilution of each serum was made in PBS containing 1% Gentamycin 50. Each serum sample was inactivated at 56°C for 30 min before testing. Twofold serum dilutions (initial dilution 1:10) were made in duplicate rows in minimum essential medium with Earle’s salts, containing 1 mg/ml Gentamycin and 5% fetal bovine serum. Equal volume of virus with a predetermined titre (100 TCID_50_ per well, 50% tissue culture infectious dose) was added and incubated at 37°C in a 5% CO_2_ gassed incubator for 1 hour. A Vero cell suspension containing 480 000 cells ml^-1^ were added in a volume of 80 μl. Plates were incubated at 37°C in 5% CO_2_ and the progress of the CPE of the virus was recorded daily for up to 4–5 days of incubation. Titres were determined as the reciprocal of the highest serum dilution that provided >50% protection of the cell monolayers.

### Cytotoxicity study

A cytotoxicity study in Vero cells was conducted to confirm the safety of the plant-produced and partially purified VP2 combinations (serotypes 1–9), without and with the addition of the adjuvants Montanide Gel 01 or Nanoalum. All at concentrations ranging from 1 μg to 2.3 ng serial dilutions per well prior to the pre-clinical trials.

### Isolation of splenocytes, antigen stimulation and flow cytometry

Mice were euthanised and their spleens aseptically collected when the trial was terminated on day 28. Spleen cells were released into incomplete RPMI media by mashing the organs and filtering through 25 mm sterile cell strainers (Fisherbrand^TM^) directly into 50 ml falcon tubes. After red blood cell lysis with ACK lysis buffer (Lonza, BP10-548E), splenocytes were re-suspended in complete RPMI-A (CP21-4418, Capricorn Scientific) media supplemented with 10% fetal bovine serum (FBS), 1% L-glutamine. Isolated cells were stimulated with either: 1) PBS or bicine buffer (mice trials 1 and 2, respectively) or 2) the relevant antigens or 3) Concanavalin A from *Canavalia ensiform*is (Jack Bean, Type IV-S) (Sigma C5275) suitable for cell culture. Stimulation of splenocytes with the plant-produced antigens (1 μg of each) were conducted as follows: trial 1, VLP/VP2 antigens of all nine serotypes combined (VLP/VP2 vaccinated group) or all nine VP2 antigens combined, for the VP2 and LAV vaccinated groups; whilst in trial 2, all nine VP2 antigens were combined to stimulate cells of LAV and VP2 vaccinated groups. Technical duplicate plates of 24 well cell culture plates (TPP) were incubated at 37°C in 5% CO_2_ for 48 hours. Unstained cells served as negative control. After 48 h of incubation, the cells were harvested by centrifugation and resuspended in 0.1% BSA/PBS with the addition of mouse Fc blocker (Biorad) for the cells to be stained.

Following antigen stimulation, cells were recovered to perform surface staining with FITC-labelled anti-CD3, PE anti-mouse CD8a and PerCP/Cyanine5.5 anti-mouse CD4 (BioLegend 100204, 100708 and 116012, respectively; all Biocom Africa). Thereafter, Brefeldin (BioLegend 420601) was added 24 hours before staining to inhibit protein transport for enhanced intracellular cytokine staining. After incubation, the cells were fixed using Cyto-Fast™ Fix/Perm Buffer Set (BioLegend 426803) followed by intracellular staining permeabilization wash buffer (BioLegend 421002). The cells were once more washed and centrifuged before resuspending in 0.1% BSA/PBS and transferred to FACS tubes. Samples were analysed with the BD FACSLyric flow cytometer (BD Biosciences) equipped with a 488 nm laser for excitation of CD3-FITC, CD8-PE, CD4-PerCP-Cy5.5. A lymphocyte gate was used during analysis to capture 15 000 cells. Data were analysed with FCSExpress version 7 (De Novo Software, Pasadena, CA, USA) and GraphPad Prism version 9 (San Diego, CA, USA). Lymphocytes were identified on a forward scatter (FSC) and side scatter (SSC) density plot. To ensure stringent single-cell gating, doublets were excluded using SSC and FSC Height and Width. Single events were gated on the FSC-H vs. FSC-W density plots.

### Statistical analysis

For statistical considerations, statistical difference at P < 0.05 (indicated with a *) were considered significant in a two tailed Student’s t test.

### Ethics approvals

Department of Agriculture, Land Reform and Rural Development (DALRRD) approvals for synthetic gene sequences (12/11/1/1/12-1377) and IFNAR mice studies (12/11/1/1/12-2932KL). The trials were conducted according to the procedures and schedule for the BSL3 facility of the Department of Science and Innovation (DSI)/ Northwest University (NWU) PCDDP, South Africa (NWU-AnimCareREC reference number NWU-00767-22-A5). All animal manipulations were performed by skilled and South African Veterinary Council (SAVC) authorised staff. Supplementary CSIR Research Ethics Committee (REC) 394/2022 approval.

## Results

### Production and purification of plant-produced AHS VLPs and VP2 antigens

**Fig 1:**
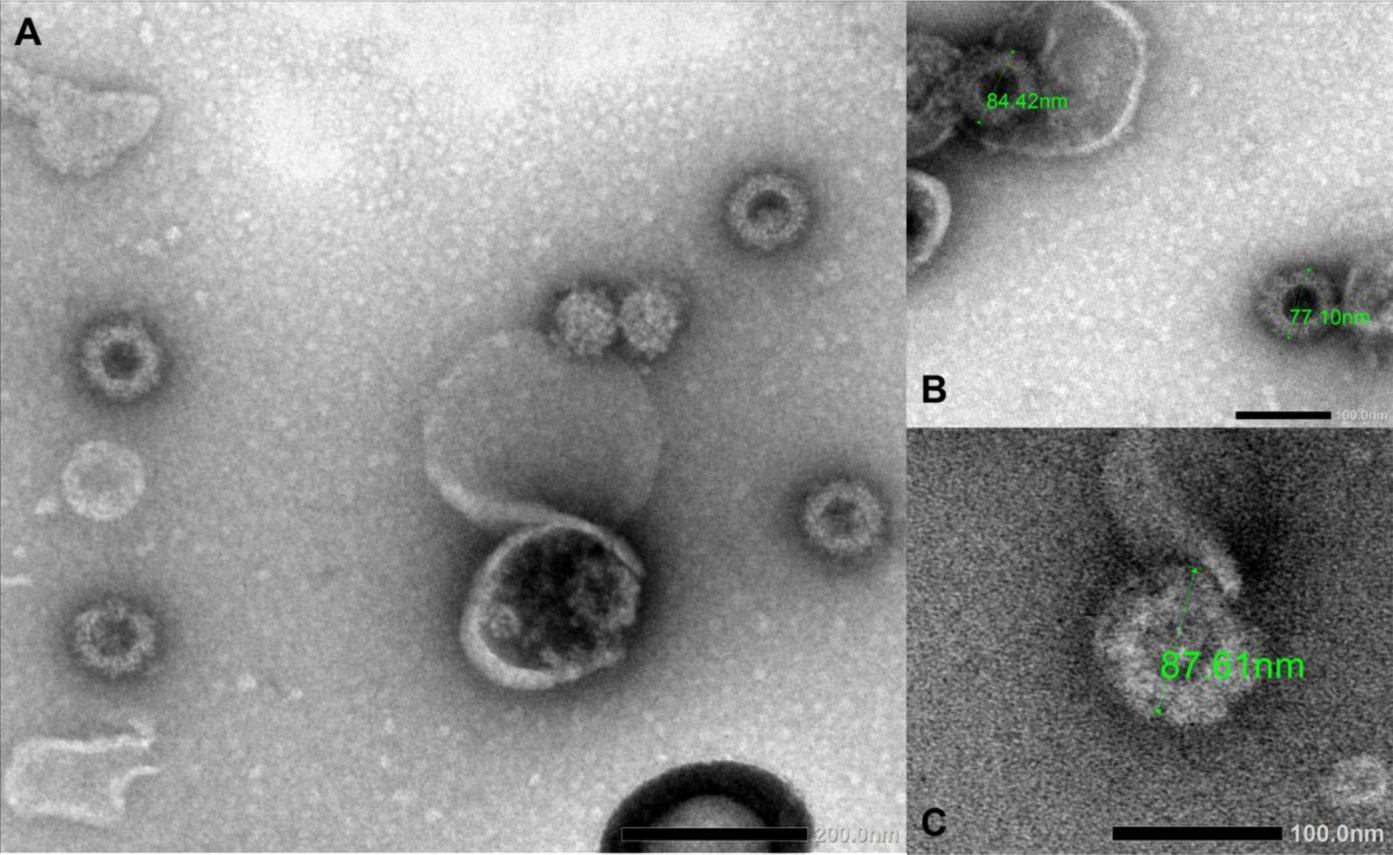
Transmission electron microscopy (TEM) images of African horse sickness VLPs. Transmission electron microscopy (TEM) images of plant-produced double chimaeric AHSV-1/5 (A, B) and AHSV-1/6 (C) VLPs. VLPs in Iodixanol fractions were stained using uranyl acetate. Fully assembled VLPs measure ∼80 nm.

**Fig 2:**
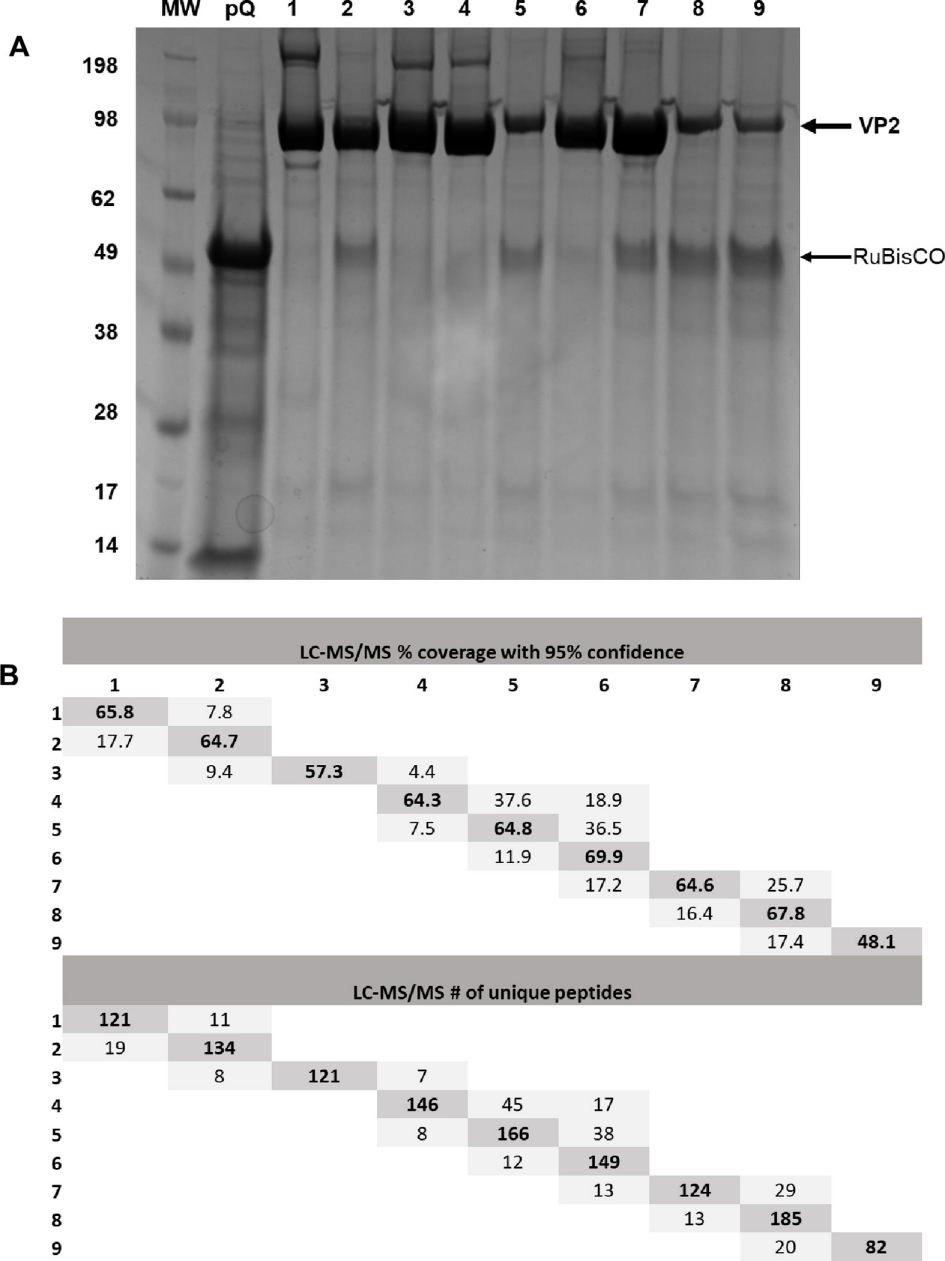
SDS-PAGE and LC-MS/MS characterization of partially purified VP2 antigens. SDS-PAGE (A) and LC-MS/MS characterization (B) of partially purified AHS VP2 proteins of all nine serotypes. Six weeks old *N*. *benthamiana* wild type were infiltrated with *Agrobacterium* AGL-1 harbouring relevant constructs to produce the individual nine VP2 fusion proteins, represented as serotypes 1–9. IMAC purified soluble VP2 and separated using a 4–12% Bolt Tris-Bis Plus precast gels (Life Technologies), and stained by Coomassie blue staining: lane MW, SeeBlue® Plus2 Pre-stained Protein Standard; lane pQ, pEAQ-HT-empty vector expressed in plant leaf tissue; lanes 1–9, IMAC partially purified VP2 fusion proteins of serotypes 1–9 respectively.

AHSV VLPs and VP2 antigens were produced in wild type *N*. *benthamiana*. In this study double chimaeric AHSV-1/5 VLPs, displaying VP2 and VP5 of serotype 5 and VP3 and VP7 of serotype 1 (VP3/VP7), measured in the order of 77 to 84 nm (Fig 1A and 1B). Similarly, the double chimaeric AHSV-1/6 VLPs assembled into particles measuring ∼87 nm but were less abundant (Fig 1C). AHSV-1/5 and AHSV-1/6 VLPs were produced at bench scale in the order of 25 and 5 mg per Kg of plant leaf tissue, respectively. The histidine-tagged AHS VP2 fusion proteins were partially purified individually and yielded at bench scale in the order of 25–35 mg per Kg of plant leaf tissue. The VP2 proteins of all nine serotypes were subjected to SDS PAGE (Fig 2A) and the appropriate bands representing VP2 (indicated with an arrow) were subjected to LC-MS/MS based peptide sequencing analysis (Fig 2B). The percentage coverage with 95% confidence of each serotype as well as the unique peptides of each serotype are depicted (Fig 2B) and clearly demonstrates the distinctness of each serotype. Ribulose-1,5-bisphosphate carboxylase/oxygenase (RuBisCO, 50 kDa) is the most abundant protein in plants as clearly demonstrated in pEAQ-HT-empty vector (pQ) expressed in plant leaf tissue but diminish when VP2 proteins are expressed (Fig 2A).

### Immune responses in IFNAR^-/-^ mice

**Fig 3:**
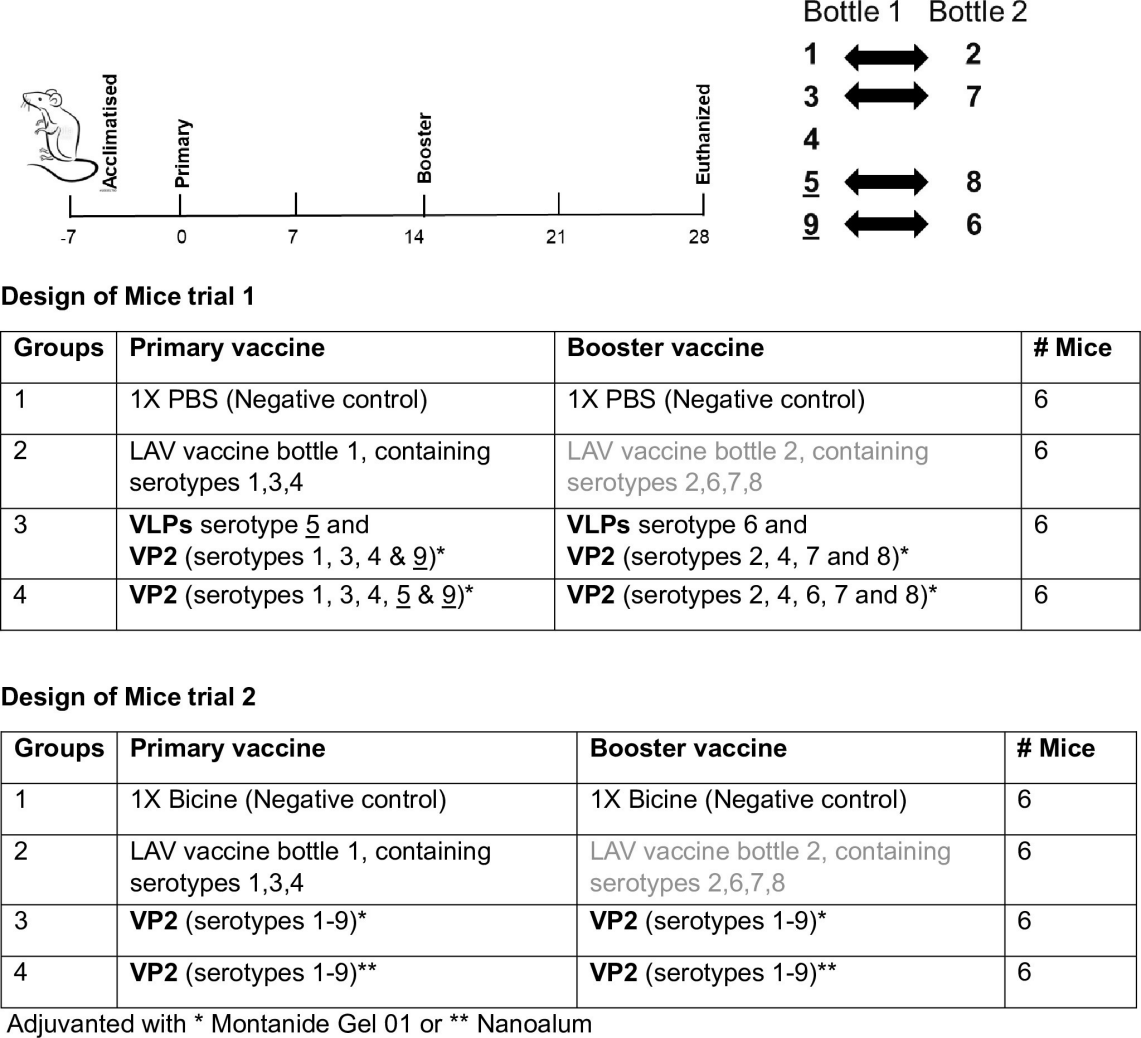
Schematic diagrams of mice trial designs. Schematic diagrams of VLP and VP2 immunogens using various prime-boost approaches and validated in IFNAR^-/-^ mice (trials 1 and 2). Proposed cross-neutralisation were indicated as previously published [21] in the commercially available LAV vaccine, bottle 1 and 2, which served as positive control vaccine. Serotypes 5 and 9 underlined as these are omitted from the LAV. Irrespective of the combinations, the plant produced VLPs or antigens were formulated as 5 μg per serotype. The commercial LAV vaccine, bottle 1, was administered as 1 μl per mouse (trial 1) and 0.2 μl per mouse (trial 2). Due to adverse effects in the mice, bottle 2 was not administered. Plant-produced vaccine products were adjuvanted with either * Montanide Gel 01 or ** Nanoalum, whereas the commercial vaccine had its own proprietary adjuvant.

The use of the commercially available and registered LAV comprises the administration of a trivalent component (serotypes 1, 3 and 4) followed by a tetravalent preparation (serotypes 2, 6, 7 and 8). In IFNAR^-/-^ mice trial 1, the plant-produced VLP/VP2 vaccine was designed to emulate this formulation, however serotypes 5 and 9 were included in the primary vaccine formulation and serotype 4 was additionally included in the booster vaccine formulation (Fig 3). Immunity against AHSV is serotype-specific with limited cross-reactivity between certain AHSV serotypes1 and 2, 3 and 7, 5 and 8, 6 and 9 (Fig 3) as reported previously [6, 21] and no notable cross-reactivity of serotype 4 to heterologous serotypes [6, 22]. Therefore, it was decided to include VP2 of serotype 4 in both primary and booster pentavalent vaccines (Fig 3, mice trial 1). Indeed, serotype 4, included in both the primary and booster VLPs/VP2 formulations (group 3), was the only plant-produced immunogen formulations that elicited antibodies to the desired titre of 1:320 in mice trial 1 whilst the neutralising antibodies for all other serotypes was ≤1:80. In the follow up mice trial, all nine serotypes were included in both the primary and booster vaccine (groups 3 and 4; mice trial 2), and adjuvanted with either Montanide Gel 01 or Nanoalum, respectively. The safety of the plant-produced VP2 product, with and without the adjuvants, were assessed in a cytotoxicity study prior to vaccination (S1 Fig).

Neutralising antibodies (nAbs) in the mice serum were determined using SNTs at the initiation (day 0) and end point (day 28) of the trial. It was confirmed that serum collected prior to inoculation (naïve mice) was free of AHS antibodies and served as negative control for each individual group. The authors previously demonstrated that plant-produced monovalent immunogens resulting in titres of 1:320, conferred protective immunity in IFNAR^-/-^ mice [8] and therefore titres of 1:320 was sought after. In this study, in mice trial 1 only serotype 4 which was included in both primary and booster vaccine resulted in titres of 1:320. In a follow up mice trial 2, where all nine serotypes (nonavalent) were included in both the primary and booster vaccine, the desired neutralising antibodies of 1:320 were obtained for serotypes 1, 4, 5, 7 and 8, whilst serotype 2, 3, 6 and 9 resulted in titres of 1:160, 1:224, 1:112 and 1:224, respectively (Table 1).

**Table 1:** Serum neutralizing assays (SNTs) of two independent mice trials.

Mice trial 1	Serotypes											
Treatment groups	1		2		3		4	5	6	7	8	9
PBS buffer negative control	0		0		0		0	0	0	0	0	0
LAV bottle 1 (serotypes 1, 3 and 4)	80		20		112		56	14	28	10	160	10
AHS VLP/VP2 pentavalent (Montanide Gel 01)	56		28		0		**320**	56	14	0	80	0
AHS VP2 pentavalent (Montanide Gel 01)	0		0		0		56	20	0	0	0	0
**Mice trial 2**	**Serotypes**											
**Treatment groups**	**1**	**2**		**3**		**4**		**5**	**6**	**7**	**8**	**9**
Bicine buffer negative control	0	0		0		0		0	0	0	0	0
LAV bottle 1 (serotypes 1, 3 and 4)	112	0		224		40		224	14	224	320	224
AHS VP2 nonavalent (Montanide Gel 01)	**>320**	160		224		**>320**		**320**	112	**320**	**320**	224
AHS VP2 nonavalent (Nanoalum)	80	0		28		112		160	14	28	112	56

Serum neutralizing test (SNT) titres of mice (pooled for each treatment group) vaccinated as detailed in Fig 3. Plant-produced immunogens representing all nine serotypes: pentavalent formulations adjuvanted with Montanide Gel 01 (trial 1) or nonavalent (all nine serotypes in a single formulation) vaccine formulations adjuvanted with either Montanide Gel 01 or Nanoalum (trial 2). The commercial LAV vaccine, bottle 1, was administered as 1 μl per mouse (trial 1) and 0.2 μl per mouse (trial 2). Due to adverse effects in the mice, bottle 2 was not administered. As expected, mice injected with either PBS or Bicine buffer remained seronegative.

Remarkably, mice vaccinated with the plant-produced VP2 antigen-based nonavalent vaccine (5 μg of each VP2) in a prime-boost regime elicited serotype specific antibodies of ≥112 to all nine serotypes (Table 1). Thus, the plant-produced nonavalent VP2 vaccine resulted in seroconversion to all nine serotypes when superior nAbs were elicited when adjuvanted with Montanide Gel 01. nAbs titres of mice vaccinated with the AHS VP2 nonavalent vaccine and adjuvanted with Montanide Gel 01 were consistently similar or superior to titres elicited by AHS LAV for each of the nine serotypes on day 28 (end point). The plant-produced nonavalent VP2 vaccine adjuvanted with Nanoalum, however, elicited nAb titres of between 0 and 160. Note, that only bottle 1 containing serotypes 1, 3 and 4 of the commercial vaccine was administered. Mice vaccinated with the LAV bottle 1 incurred adverse effects in the mice, and for humane purposes, bottle 2 (containing serotypes 2, 6, 7 and 8) were not administered. As expected, mice injected with buffer alone remained seronegative.

### Isotyping of immune responses in IFNAR^-/-^ mice

**Fig 4:**
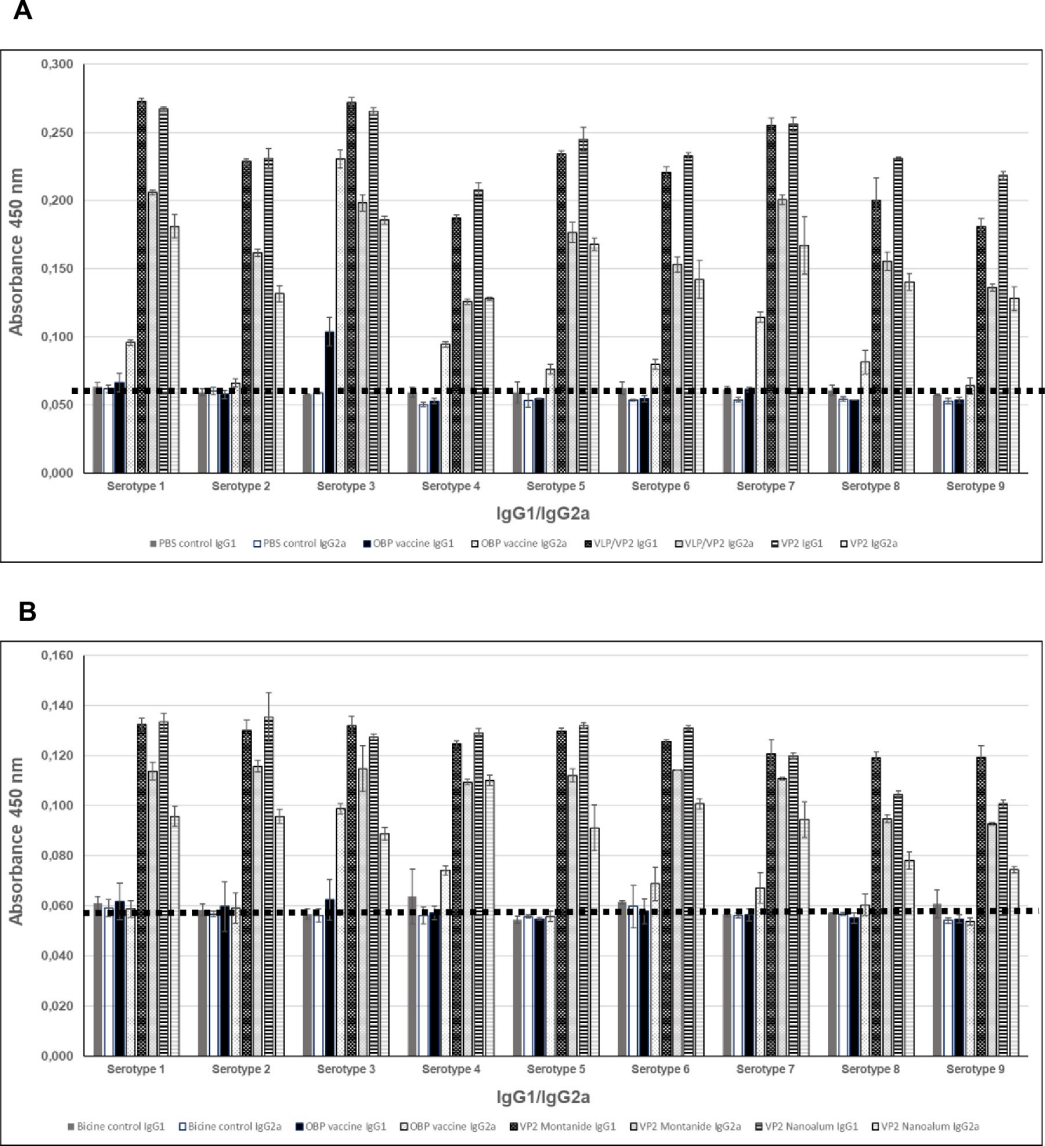
ELISA detection and isotyping of mice serum at experimental endpoint. ELISA detection and isotyping of mice serum collected at the experimental endpoint. Serum of the six mice in each group were pooled unless otherwise stated. (A) IFNAR trial 1: mice were vaccinated with PBS buffer as negative control (group 1), the commercial LAV vaccine (group 2, 4 mice serum pooled, 2 succumbed before the end point), the plant produced VLP/VP2 (group 3) or plant produced VP2 pentamer prime-boost vaccines (group 4) with groups 3 and 4 adjuvanted with Montanide Gel 01. (B) IFNAR trial 2: mice were vaccinated with Bicine buffer as negative control (group 1), the commercial LAV vaccine (group 2, serum of a single mice, 5 succumbed before the end point), the plant produced multivalent VP2, serotypes 1–9 prime-boost vaccine adjuvanted with either Montanide Gel 01 (group 3) or Nanoalum (group 4). Triplicate technical replicate samples for each bar and timepoint. Single serotype VP2 (1 μg per well) were used to coat the ELISA plates.

IgG2a/IgG1 isotyping reflects the balance of T_h_1-type/T_h_2-type immune responses to some degree. Higher IgG2a mediates strong cell-mediated cytotoxicity (ADCC) effect and opsonophagocytosis by macrophages. This immune response was observed when mice was vaccinated with the commercially available vaccine, primary vaccine (bottle I, serotypes 1,3,4, group 2) which is indicative of a stronger bias towards T_h_1 responses for serotype 1, 3 and 4 (Fig 4) and even serotype 7, probably due to potential cross reactivity of serotypes 3 and 7. In contrast, all mice immunized with plant-produced VLP/VP2 or VP2 vaccines formulated in water-in-oil Montanide or Nanoalum adjuvants had IgG1:IgG2a ratios >1, indicating that these adjuvants induced primarily Th_2_-type antibody responses (Fig 4).

### T-cell responses in IFNAR^-/-^ mice

**Fig 5:**
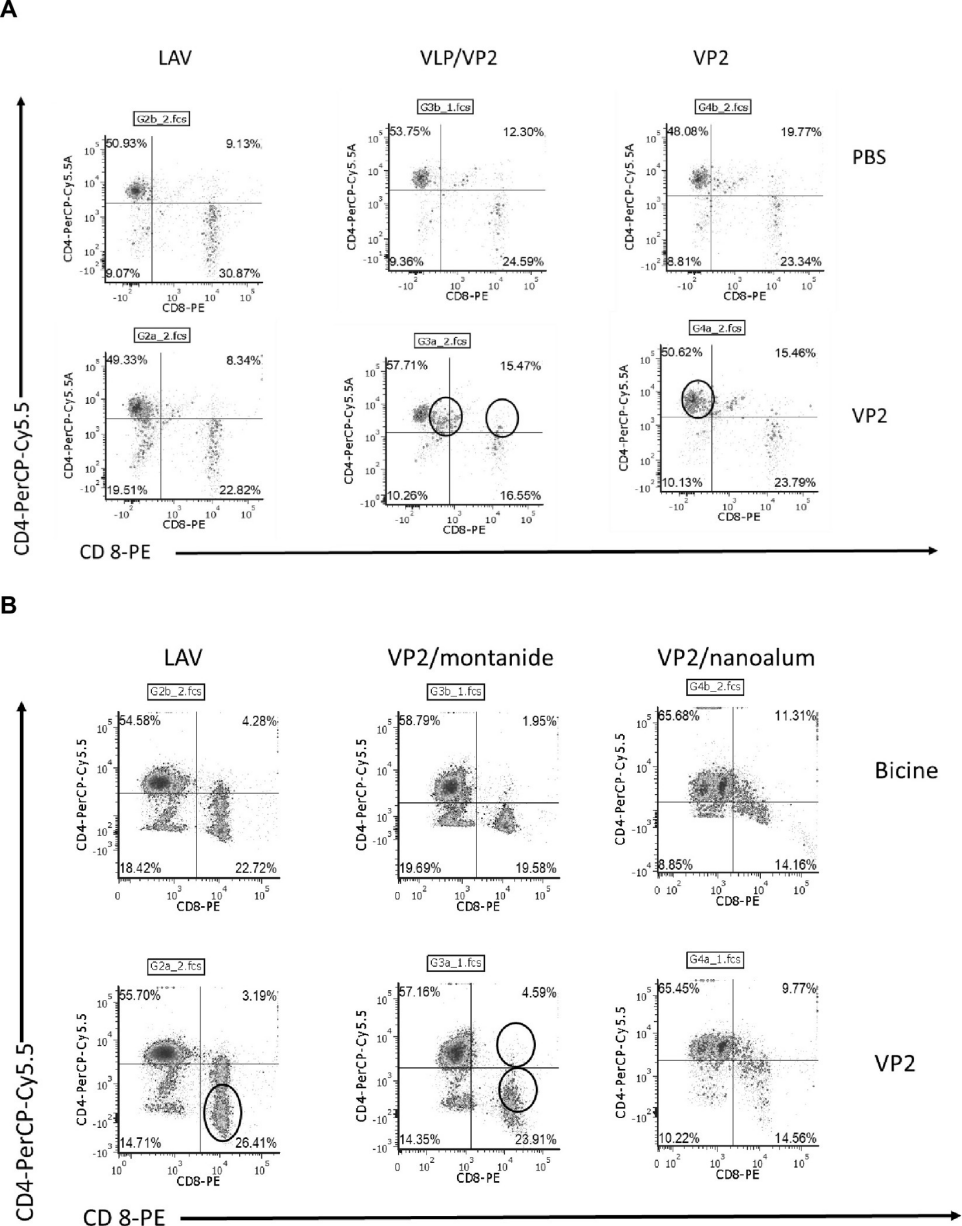
T-cell responses of mice splenocytes at the experimental endpoint. T-cell responses of mice splenocytes (mice spleens pooled per treatment group) harvested 14 days after booster vaccination with different formulations of the vaccine. Splenocytes stained with CD4- and CD8-specific antibodies and analysed by flow cytometry. Representative flow cytometry plots are illustrated. The % splenocytes positive for both CD4+ and CD8+ were quantified and presented in the quadrants A: IFNAR trial 1: mice were vaccinated with either the commercial LAV vaccine, the plant produced VLP/VP2, or plant produced VP2 pentamer prime boost vaccines both adjuvanted with Montanide Gel 01. B: IFNAR trial 2: mice were vaccinated the commercial LAV vaccine, the plant produced multivalent VP2, serotypes 1–9 prime-boost vaccine adjuvanted with either Montanide Gel 01 or Nanoalum.

Splenocytes from donor mice (mice spleens pooled per treatment group) were isolated and stimulated for 48 hours with the relevant antigens to measure the cellular immune responses by means of flow cytometry (Fig 5A and 5B). Compared to unstimulated PBS controls of the same mice, the LAV vaccine showed a measure of CD8+ stimulation (22.7% to 26.41%) only in the second study in the mice as expected (Fig 5B). When the plant-produced groups were analysed compared to the unstimulated PBS and Bicine controls, the VLP/VP2 regimen resulted in CD4+ stimulation (53.57% to 57.71%, Fig 5A) and CD4+/CD8+ stimulation (12.3% to 15.47%, Fig 5A) which is required to induce cell memory. A similar observation was also made in a previous study on this combination of antigens [8]. In this study, primary (serotypes 1, 3–5 and 9) and booster (serotypes 2, 4, 6–8) pentavalent vaccinations showed, apart from serotype 4, a poor response when SNTs were measured of all nine serotypes (Table 1, mice trial 1) despite the double positive stimulation of the VLP/VP2 vaccinated group. This provided the motivation to include all 9 serotypes in both the primary and booster vaccinations (mice trial 2). A double positive response for CD4+/CD8+ stimulation (1.95% to 4.59%) was thereafter observed for the group that also included the Montanide gel 01 as adjuvant (Fig 5B).

## Discussion

Immunity against AHSV is serotype-specific and protection against all nine AHSV serotypes is imperative in an endemic setting [6]. As VP2 is the most variable protein, AHS VP2-based vaccine candidates have been sought after globally for almost three decades. These candidates include insect cell produced VP2 as well as recombinant canarypox and modified Vaccinia Ankara (MVA) viral vectored vaccines [3, 4, 6, 15, 16, 18, 23–26]. As an alternative approach, we produced VP2 proteins in plants, mitigating the risk of eliciting pre-existing immunity to either the canarypox or modified vaccinia Ankara vector backbones. This is the first study where plant-produced AHS VP2 fusion proteins of all nine serotypes were included in multivalent vaccine formulations resulting in antibody titres ranging from 1:112 to 1:320 for each serotype complimented by T-cell responses, double positive CD4+/CD8+ stimulation (1.95% to 4.59%) which is required to induce cell memory.

Previously, the authors demonstrated good immune responses when IFNAR^-/-^ mice [8] and horses [12] were vaccinated with plant-produced AHS VLPs. VLP technology seeks to harness the optimally tuned immunostimulatory properties of natural viruses whilst omitting the infectious trait [27] resulting in highly immunogenic subunit vaccines. It is anticipated that single antigen-based subunit vaccines do not have these immunostimulatory properties but with appropriate adjuvants might perform similarly. Indeed, we demonstrated that AHS VLPs and recombinant VP2 protein conferred protective immunity in IFNAR^-/-^ mice equally well when adjuvanted with an oil-in-water based Montanide Gel 01 [8].

Calvo-Pinilla and co-workers [16] indicated that protection against AHSV induced by MVA-VP2 vaccination is mediated primarily by antibodies in IFNAR^-/-^ mice and that the level of protection observed correlated with antibody titre [16]. Nevertheless, the required titres to confer protective immunity against AHSV in horses is not established [15]. The commercially available vaccine manufacturer relies on the induction of a titre of 1:16 as minimum requirement for protection in horses [28] whilst other data suggest that a titre of 1:64 [29] would confer solid immunity against AHSV with prevention of clinical signs and viraemia in horses. Van Rijn and co-workers [29] demonstrated that horses vaccinated with freshly formulated undiluted inactivated AHSV (equivalent of ± 10^7.5^ TCID_50_/ml) vaccine and adjuvanted with 10% Montanide Pet Gel A, survived AHSV challenge at titres of 64, although they developed viremia and fever for one or more days. Thus, van Rijn and co-workers [29] estimated that a threshold of the nAb titre for protection in horses was approximately 64. Thus, we considered titres of ≥64 as indicative of seroconversion in mice. In this mice trial, a nonavalent VP2 antigen-based vaccine (5μg dose of VP2 of each serotype) administered as primary and booster vaccine two weeks apart, resulted in end point antibody titres of ≥112 for each of the nine serotypes when adjuvanted with Montanide Gel 01. Should a titre of 16 be sufficient, the plant-produced VP2 antigen-based vaccine adjuvanted with Nanoalum, resulting in titres of 14 to 160, also has the potential to confer protection. It is interesting that the primary commercial vaccine containing only serotypes 1, 3 and 4, resulted in seroconversion of serotypes 1 and 3 as expected but also serotypes 5, 7, 8 and 9. Seroconversion to serotype 7 can be understood in the light of the genetic relatedness and potential cross-neutralisation of serotype 3 and 7. However, seroconversion of mice vaccinated with the AHS LAV bottle 1 only (containing serotypes 1, 3 and 4) to serotypes 5, 8 and 9 was not expected. Nevertheless, although the duration of the immunity induced by these antigens still needs to be determined in the target animals, horses, the data clearly demonstrate the potential of this candidate plant-produced VP2 antigen-based vaccine.

Ratios of IgG1 to IgG2a antibody isotyping has been used as an indirect measure of T helper 1 (T_h_1) and T helper 2 (T_h_2) types of immune responses. Huber and co-workers [30] (2006) reported that increased expression of vaccine induced IgG2a antibodies coincided with clearance of virus and increased protection against a lethal influenza challenge but argues for the stimulation of both IgG1 and IgG2a in the development of vaccine regimens. In this study, we demonstrated using an in-house ELISA assay that plant-produced vaccines, whether VLP/VP2 combinations or VP2 alone, elicit a higher IgG1 versus IgG2a for all nine serotypes. The absorbance values at OD_450_ are higher and most often double the values obtained when mice were vaccinated with the commercial vaccine. Yet, mice vaccinated with the commercial vaccine only received the primary vaccine (containing serotypes 1,3 and 4) in the second trial. This is because two of the six mice in group 2 succumbed (trial 1) and four of the six in group 2 succumbed in the second trial. The booster (bottle 2, containing serotypes 2, 6, 7 and 8) was never administered in the second trial. An interesting observation was the detection of IgG2a of serotype 7 in mice vaccinated only with bottle 1 (containing serotypes 1, 3 and 4). This is probably the result of the genetic relatedness and potential cross-neutralisation of serotype 3 and 7. Although these pilot studies were exclusively immunogenicity studies, the analyses of different serum antibody isotypes after vaccination with either VLP/VP2 or VP2 antigen-based vaccines, and two different adjuvants, strengthen the argument that vaccine induced IgG1 and IgG2a antibodies may potentially contribute to protective immunity.

DIVA compliance, the ability to differentiate between naturally infected and vaccinated horses, is imperative to confirm virus-free status of horses for export to non-endemic countries; movement to AHSV free regions in endemic countries and serve in early detection of outbreaks to contain spread [8]. A low cross-neutralising antibody titre was obtained when guinea pigs were vaccinated with insect cell produced AHS VP2-based vaccines at 10–12.5 μg VP2 per serotype for genetically related serotypes [4]. Immunization of guinea pigs with VP2 cocktails also triggered neutralising antibodies albeit at lower titres (4–117) to each of the serotypes in the cocktail. In this study, it was demonstrated that the plant-produced multivalent vaccine containing partially purified VP2 of each of the 9 AHSV serotypes at 5 μg per serotype not only elicits desirable titres (>112) to all the serotypes but serves equally well in in-house ELISA-based diagnostics. It is known that AHS VP2 is a serotype-specific capsid antigen of AHSV that forms the basis of ELISA-based serological diagnostic tests [16]. Yet, non-scalable purification processes required to eliminate non-specific binding of plant proteins and the magnitude of losses during purification hinder the use of the plant-produced VP2 for commercially available diagnostics at present.

Ideally, the plant-produced VLP/VP2 combination or VP2 antigen-based vaccine should align with the main standards for modern veterinary vaccines i.e. safety, efficacy, DIVA compliance, acceptance and affordability [31]. Indeed, the plant-produced vaccine candidates have the potential to meet all these criteria. In this study, the safety of the plant-produced vaccines was demonstrated, and titres indicative of protective immunity obtained in mice. As the plant produced VP2 antigen vaccine is void of VP7, horses vaccinated will not seroconvert to the well-established ELISA test kit based on VP7 antibodies and renders the VP2 vaccine DIVA compliant. The ease of production and purification of the plant-produced VP2 proteins of all nine serotypes, underpin affordability for sport horses as well as rural community draft horses. The approval of a plant-produced vaccine product for human health, Medicago’s Covifenz^®^ authorised by Health Canada, paves the way for Biopharmed vaccine antigens for animal health.

AHS is a highly fatal disease of horses, and horse farming in Africa could not have been possible without the use of live attenuated vaccines [6]. Yet, a safe, efficacious, DIVA compliant, globally accepted, and affordable vaccine has been elusive up until now. Although a multivalent vaccination strategy using baculovirus expressed VP2-based vaccines was previously pursued in guinea pigs [4], here we demonstrate the ability of a nonavalent plant produced AHS VP2 antigen-based vaccine to enable seroconversion in IFNAR^-/-^ mice to all nine AHSV serotypes. The plant-produced VP2-based vaccination not only shows promise to prevent disease in an endemic and non-endemic country but also has diagnostic potential enabling movement of equids between different AHSV controlled geographical regions as well as international trade of horses from the African continent.

## Conclusion

Plant biopharming is acknowledged for its scalability, speed, versatility and low production costs and is an increasingly promising molecular pharming platform for both human and veterinary vaccines [32]. In this study, we demonstrated *N*. *benthamiana* plant production and bench scale purification (25–35 mg per Kg of plant leaf tissue) of AHSV VP2 antigens from nine serotypes which were then formulated as nonavalent vaccine candidates. The nonavalent VP2 antigen-based vaccine was demonstrated to be highly immunogenic and has the potential to open frontiers for a safe and highly efficacious AHS vaccine suitable not only for Sub-Saharan Africa but also for the world. The ease and speed of production may also facilitate rapid response production of safe and efficacious mono- and bivalent vaccine formulations suitable for localised outbreaks in endemic and non-endemic countries world-wide. Immunogenicity studies in the target animals, horses, are underway.

## Supporting information

S1 Fig Cytotoxicity assessment of plant-produced immunogens. Cytotoxicity studies in Vero cells validating the (A) adjuvanted commercial vaccine, (B) VP2 proteins nonavalent (serotypes 1–9) combined versus plant extract, (C) plant produced VP2 proteins nonavalent (serotypes 1–9) combined and adjuvanted with Montanide Gel 01 or (D) plant produced VP2 proteins nonavalent (serotypes 1–9) combined and adjuvanted with Nanoalum. Statistical difference at P < 0.05 (indicated with a *) were considered significant in a two tailed Student’s t test.
(PDF)

S1 Raw images (PDF)
