# Supplementary material for: Immunogenic profile of a plant-produced nonavalent African horse sickness viral protein 2 (VP2) vaccine in IFNAR-/- mice
Source: PLoS One. 2024 Apr 16;19(4):e0301340. doi: 10.1371/journal.pone.0301340 (PMC11020708; doi:10.1371/journal.pone.0301340)
Supplement: S1 Fig — Cytotoxicity studies in Vero cells validating the (A) adjuvanted commercial vaccine, (B) VP2 proteins nonavalent (serotypes 1–9) combined versus plant extract, (C) plant produced VP2 proteins nonavalent (serotypes 1–9) combined and adjuvanted with Montanide Gel 01 or (D) plant produced VP2 proteins nonavalent (serotypes 1–9) combined and adjuvanted with Nanoalum. Statistical difference at P < 0.05 (indicated with a *) were considered significant in a two tailed Student’s t test. (PDF) [file pone.0301340.s001.pdf]

A

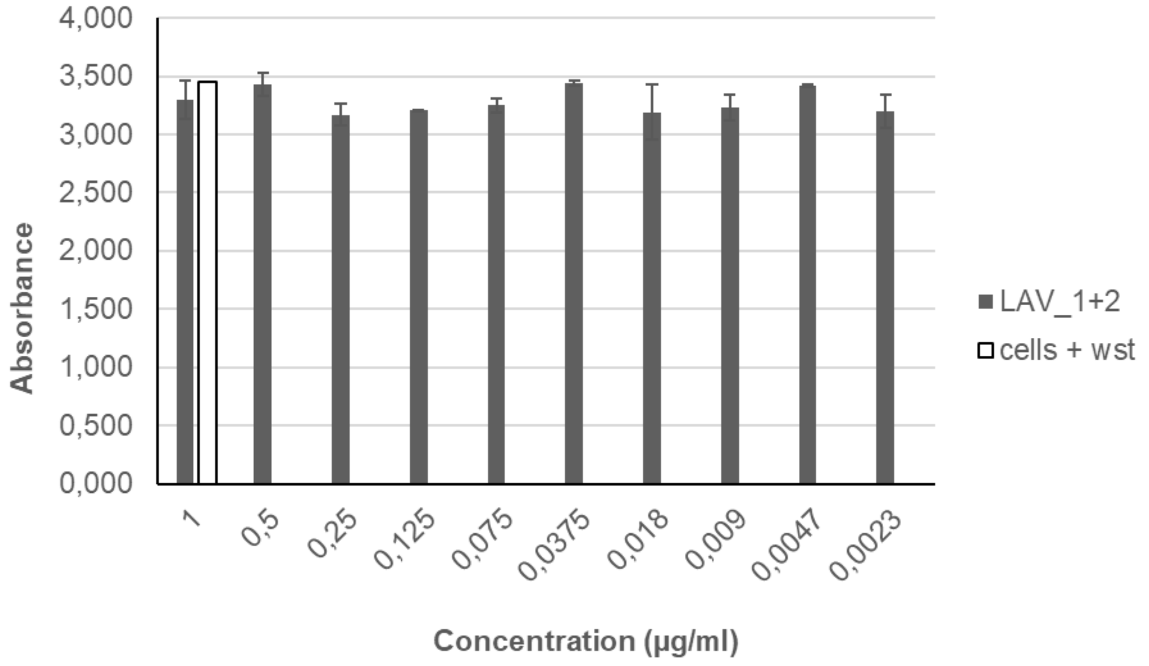
4,000
3,500
3,000
2,500
2,000
1,500
1,000
0,500
0,000
Absorbance
1
0,5
0,25
0,125
0,075
0,0375
0,018
0,009
0,0047
0,0023
Concentration (µg/ml)
LAV_1+2
cells + wst

B

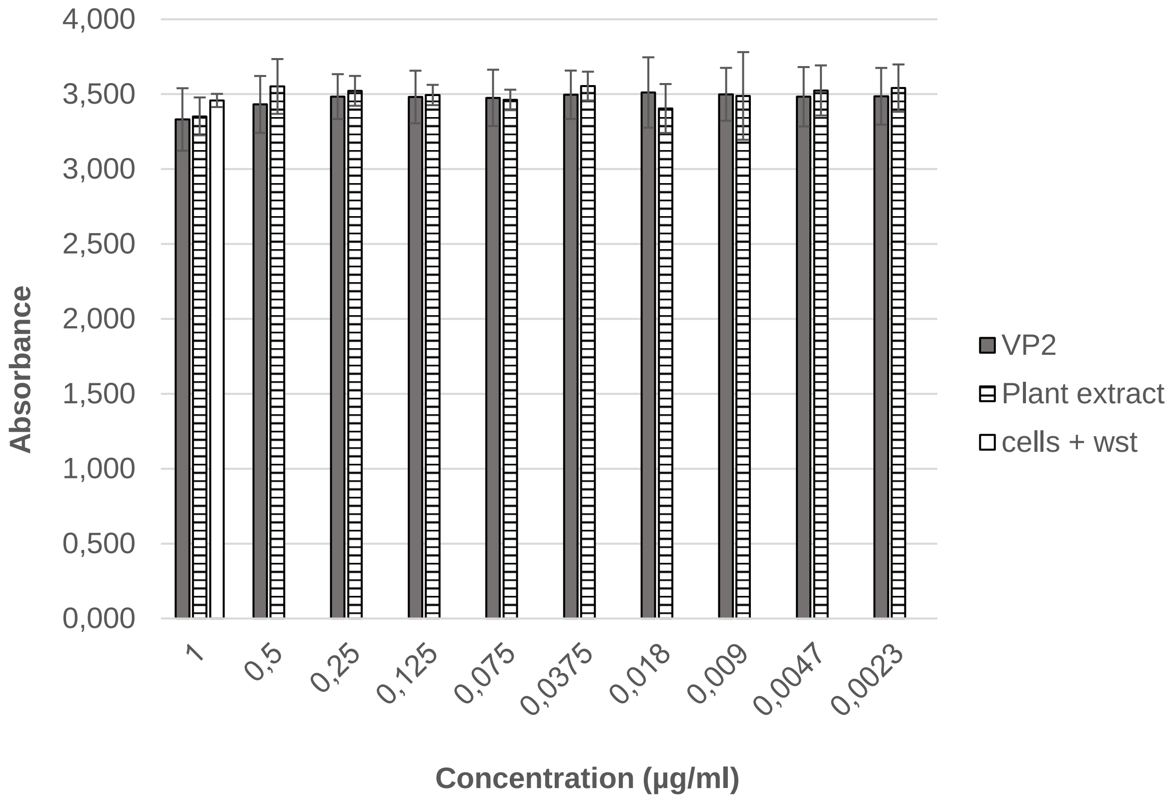
4,000
3,500
3,000
2,500
2,000
1,500
1,000
0,500
0,000
Absorbance
1
0,5
0,25
0,125
0,075
0,0375
0,018
0,009
0,0047
0,0023
Concentration (µg/ml)
VP2
Plant extract
cells + wst

C

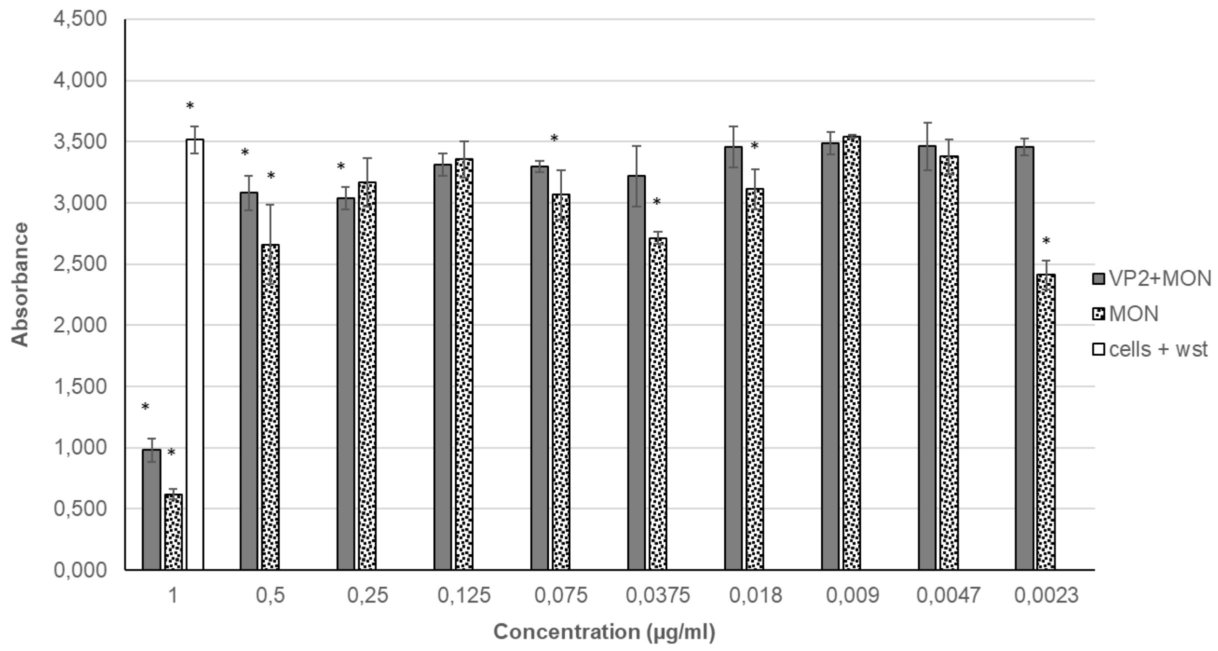

T-test	1	0,5	0,25	0,125	0,075	0,0375	0,018	0,009	0,0047	0,0023
VP2+MON vs MON	0,040	0,236	0,355	0,720	0,226	0,074	0,064	0,490	0,646	0,009
VP2 vs cells + wst	0,000	0,029	0,005	0,120	0,083	0,132	0,643	0,786	0,697	0,564
MON vs cells + wst	0,000	0,021	0,055	0,214	0,028	0,003	0,024	0,743	0,308	0,002

D

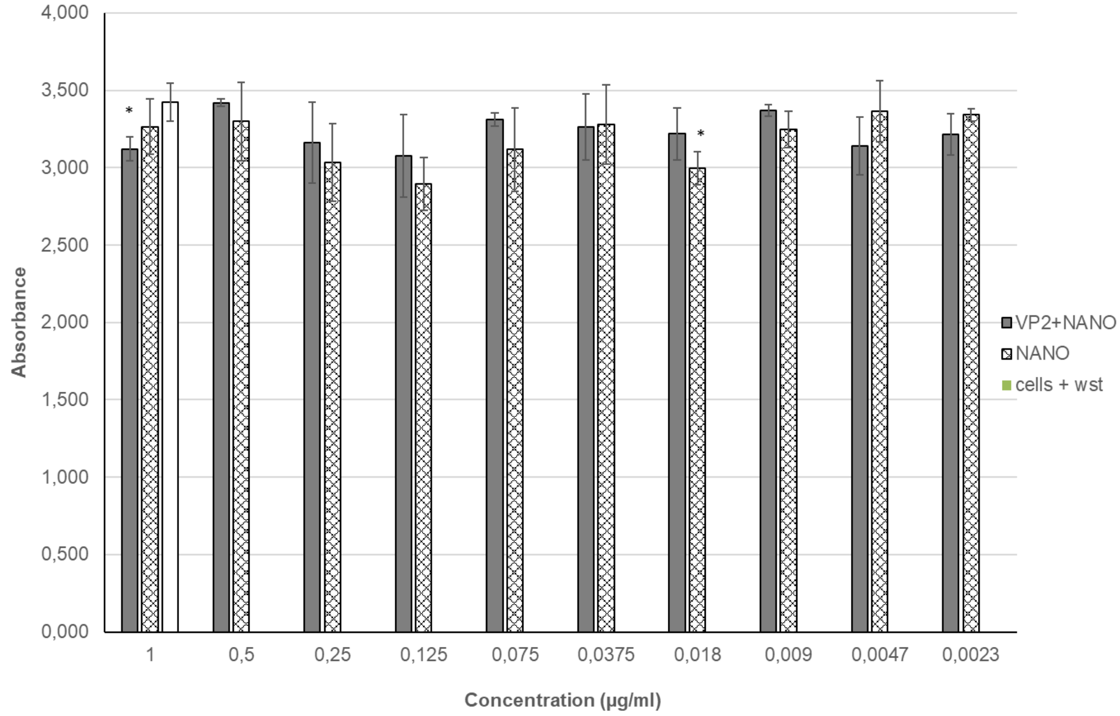

T-test	1	0,5	0,25	0,125	0,075	0,0375	0,018	0,009	0,0047	0,0023
VP2+NANO vs NAI	0,262	0,565	0,573	0,377	0,290	0,942	0,124	0,164	0,227	0,304
VP2+NANO vs cell	0,022	0,972	0,192	0,111	0,199	0,323	0,162	0,487	0,094	0,117
NANO vs cells + ws	0,272	0,492	0,073	0,012	0,147	0,424	0,010	0,143	0,684	0,444

Figure S1: Cytotoxicity studies in Vero cells validating A) adjuvanted commercial vaccine, B) plant extract, plant produced VP2 proteins nonavalent (serotypes 1-9), adjuvanted with either C) Montanide Gel 01 or D) Nanoalum.
