# Supplementary material for: Immunogenic profile of a plant-produced nonavalent African horse sickness viral protein 2 (VP2) vaccine in IFNAR-/- mice
Source: PLoS One. 2024 Apr 16;19(4):e0301340. doi: 10.1371/journal.pone.0301340 (PMC11020708; doi:10.1371/journal.pone.0301340)
Supplement: S1 Raw images — (PDF) [file pone.0301340.s002.pdf]

Image for PLOS ONE below

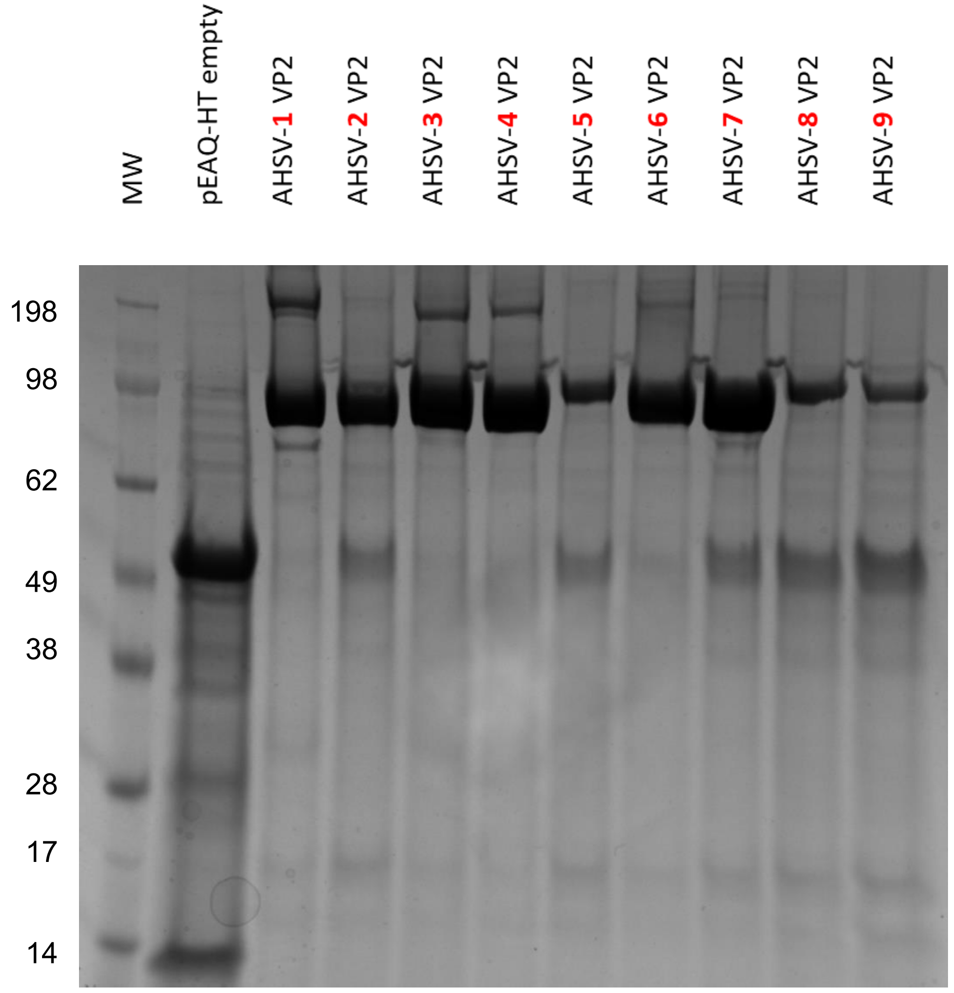

Alternative images of various batches below demonstrating the same trend of purified VP2 proteins and validity of the SDS-PAGE images (some uncropped). Although the concentrations of the AHS VP2 proteins of each individual serotype (serotypes 1-9) differ, the trend of consistently producing and purifying the AHS VP2 proteins remain the same.

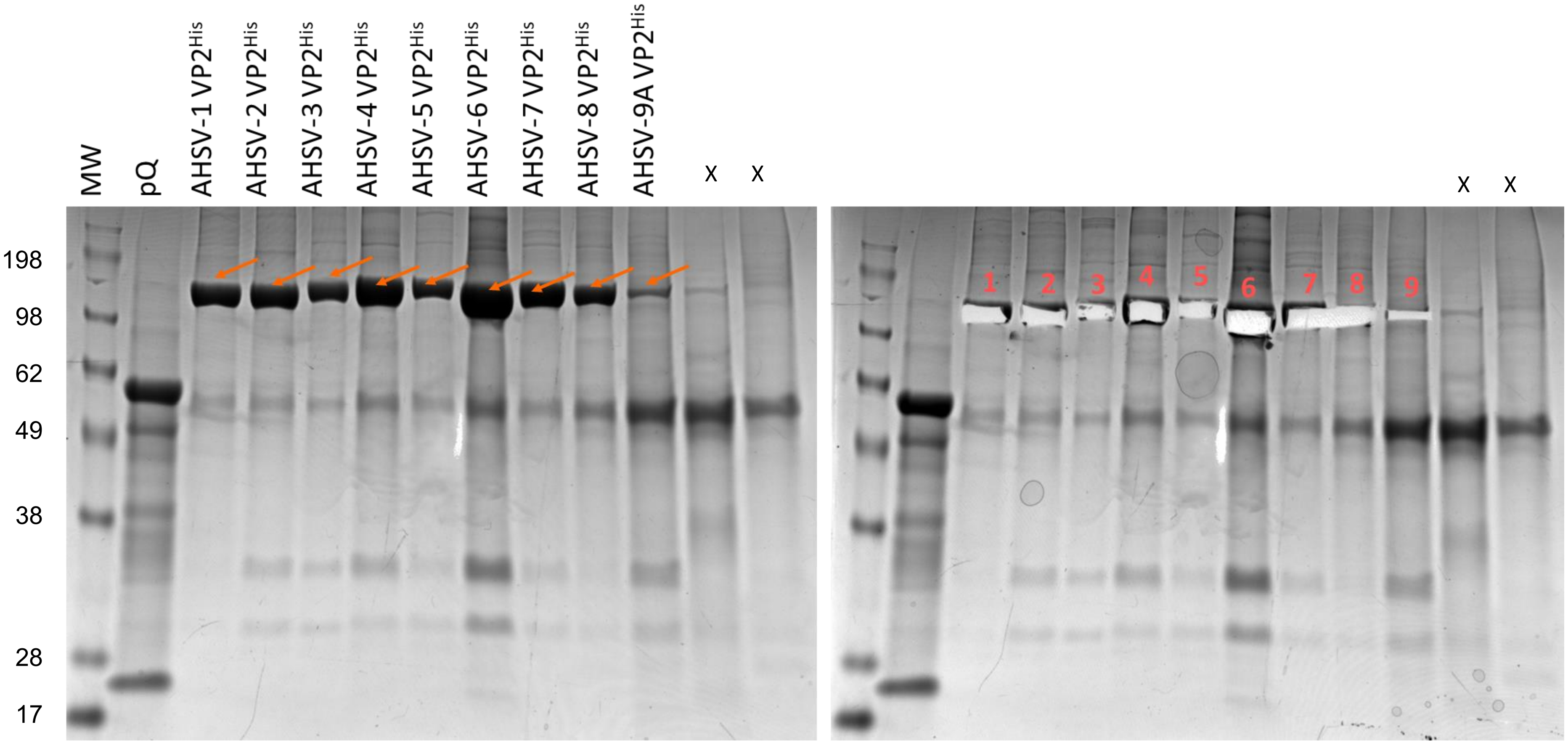

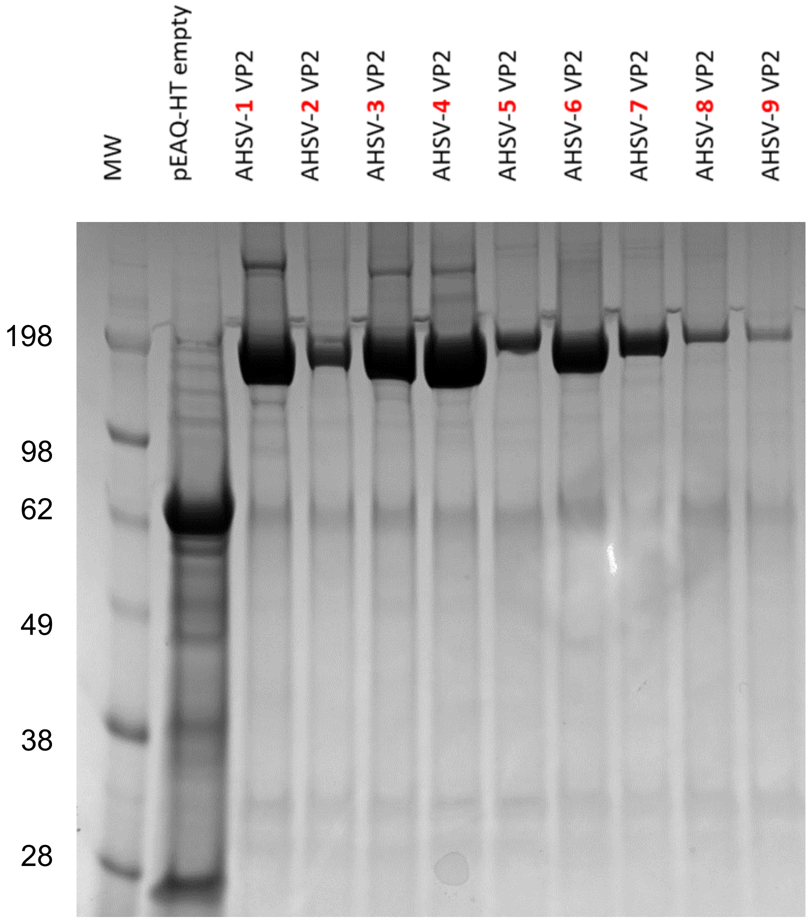

MW
pEAQ-HT empty
AHSV-1 VP2
AHSV-2 VP2
AHSV-3 VP2
AHSV-4 VP2
AHSV-5 VP2
AHSV-6 VP2
AHSV-7 VP2
AHSV-8 VP2
AHSV-9 VP2
198
98
62
49
38
28
